# Glaucocalyxin A Attenuates Allergic Responses by Inhibiting Mast Cell Degranulation through p38MAPK/NrF2/HO-1 and HMGB1/TLR4/NF-*κ*B Signaling Pathways

**DOI:** 10.1155/2021/6644751

**Published:** 2021-04-30

**Authors:** Yihua Piao, Jingzhi Jiang, Zhiguang Wang, Chongyang Wang, Shan Jin, Li Li, Liangchang Li, Hongmei Piao, Zhehu Jin, Lianhua Zhu, Guanghai Yan

**Affiliations:** ^1^Jilin Key Laboratory for Immune and Targeting Research on Common Allergic Diseases, Yanbian University, Yanji 133000, China; ^2^Department of Intensive Care Unit, Affiliated Hospital of Yanbian University, Yanji, 133000, Jilin, China; ^3^Department of Anatomy, Histology and Embryology, Yanbian University Medical College, Yanji 133002, China; ^4^Department of Respiratory Medicine, Yanbian University Affiliated Hospital, Yanji 133000, China; ^5^Department of Dermatology, Yanbian University Hospital, Yanji 133002, China

## Abstract

Glaucocalyxin A (GLA) has various pharmacological effects like antioxidation, immune regulation, and antiatherosclerosis. Here, in this study, the effect and mechanism of GLA on mast cell degranulation were studied. The results of the anti-DNP IgE-mediated passive cutaneous anaphylaxis (PCA) showed that GLA dramatically inhibited PCA in vivo, as evidenced by reduced Evans blue extravasation and decreased ear thickness. In addition, GLA significantly reduced the release of histamine and *β*-hexosaminidase, calcium influx, cytokine (IL-4, TNF-*α*, IL-1*β*, IL-13, and IL-8) production in the RBL-2H3 (rat basophilic leukemia cells), and RPMCs (peritoneal mast cells) in vitro. Moreover, we further investigated the regulatory mechanism of GLA on antigen-induced mast cells by Western blot, which showed that GLA inhibited Fc*ε*RI-mediated signal transduction and invalidated the phosphorylation of Syk, Fyn, Lyn, Gab2, and PLC-*γ*1. In addition, GLA inhibited the recombinant mouse high mobility group protein B1- (HMGB1-) induced mast cell degranulation through limiting nuclear translocation of NF-*κ*Bp65. Treatment of mast cells with siRNA-HMGB1 significantly inhibited HMGB1 levels, as well as MyD88 and TLR4, decreased intracellular calcium levels, and suppressed the release of *β*-hexosaminidase. Meanwhile, GLA increased NrF2 and HO-1 levels by activating p38MAPK phosphorylation. Consequently, these data suggest that GLA regulates the NrF2/HO-1 signaling pathway through p38MAPK phosphorylation and inhibits HMGB1/TLR4/NF-*κ*B signaling pathway to reduce mast cell degranulation and allergic inflammation. Our findings could be used as a promising therapeutic drug against allergic inflammatory disease.

## 1. Introduction

Mast cells induce allergic inflammation through the secretion of inflammatory mediators [1], and a variety of cell membrane receptors are expressed on their surface. Among them, the combination of IgE and Fc*ε*RI and the crosslinking of Fc*ε*RI and multivalent antigens cause degranulation of mast cells, which in turn leads to the release of a large number of inflammatory mediators, including secreted granules (containing histamine and proteases), cytokines (such as TNF-*α* and IL-13), growth factors, and chemokines [2], which potentiate inflammatory immune responses via the secretion of cytokines [3]. Nrf2 is an important transcription factor in the cap'n'collar family. It is activated when stimulated by oxidative stress and binds to the cis-acting element of the antioxidant response element to initiate the expression of downstream related antioxidant enzymes, such as superoxide dismutase, catalase, and heme oxygenase (HO-1) [4]. Among them, HO-1 downregulates the inflammatory response by degrading inflammatory heme compounds and bilirubin [5].

High mobility group protein B1 (HMGB1) belongs to the HMG family and is a nonhistone nucleoprotein present in various tissues [6]. HMGB1 plays an important role in allergic diseases. For example, interference with the HMGB1/TLR4 signaling pathway suppresses airway inflammation in asthma [7]. However, the role of HMGB1 in mast cells is rarely reported.

Glaucocalyxin A (GLA) is a diterpenoid with an enantiomeric 15-oxo-16-kaurene backbone structure. It has various pharmacological effects, such as antiarteriosclerosis, antitumor, anticoagulation, antithrombosis, antioxidation, antibacterial effect, and immunosuppression [8, 9]. However, its function in allergic diseases has been poorly studied. Here, we explored the effect and mechanism of GLA on a hypersensitive mouse model *in vivo* and *in vitro*.

## 2. Materials and Methods

### 2.1. Animals

BALB/c mice (*n* = 40, aged 4 to 6 weeks, weighing (18 ± 5) g) and male Sprague–Dawley rats (*n* = 20) were purchased from the Health Science Center of Yanbian University (Yanji, China). All animals were housed under conditions of temperature 22 ± 2°C and relative humidity 55 ± 5%, with a 12 h light-dark cycle. All animal experiments were conducted according to the ethical guidelines of the Ethics Committee of Yanbian University (SCXK (Ji) 2017–0003). The “Regulations on the Administration of Laboratory Animals” were followed to reduce the suffering of mice.

### 2.2. Passive Cutaneous Anaphylaxis (PCA) Test

Mice were sensitized by intradermal injection of 0.5 *μ*g of anti-DNP IgE in 50 *μ*L PBS into the ear. After 48 h, 10, 20, and 40 mg/kg of GLA were orally administered. And 1 h later, each mouse was injected with a mixture of DNP-HSA (0.1 mg/mouse) and 4% Evans blue (Unchem, Shanghai, China) (1 : 1) into the tail vein. After 30 min, the ears were collected for dye staining and histology analysis. Ear thickness was measured with a digital micrometer (Kawasaki, Japan). The Evans blue dye was extracted after incubation with 1 mL formamide for 24 h at 55°C. The absorbance of the dye was measured at 620 nm on Spectra MAX PLUS (Molecular Devices, Sunnyvale, CA, USA).

### 2.3. Histology Analysis and Mast Cell Counting

Ears were fixed in 4% formaldehyde, embedded in paraffin, and cut into 5 *μ*m sections. The tissue sections were then subjected to hematoxylin-eosin and toluidine blue (Sigma, USA) staining. Mast cell counts were performed by randomly selecting five fields at 100× magnification.

### 2.4. Preparation, Culture, and Treatment of Mast Cells

The isolation and purification of RPMCs from rats were performed using methods described previously [10, 11]. The isolated RPMCs and RBL-2H3 cells (Fuheng Biology, Shanghai) were cultured in DMEM (Thermo Fisher Scientific, Waltham, MA, USA) containing 10% FBS, 100 U/ml sodium penicillin, and 100 *μ*g/ml streptomycin in an incubator at 37°C, 5% CO_2_.

RBL-2H3 cells and RPMCs were divided into three groups of control, IgE + Ag, and IgE + Ag + GLA groups. Control groups were cultured normally without any drug treatment. Cells in the IgE + Ag group were treated with 50 ng/ml of anti-DNP IgE (Sigma, USA) for 6 h and then with 100 ng/ml of DNP-HSA (Sigma, USA) for 10 min to induce mast cell degranulation. Cells in IgE + Ag + GLA were treated with 50 ng/ml of anti-DNP IgE for 6 h, different concentrations of GLA (purity ≥ 98%; Yuanye Biotech., Shanghai, China) for 30 min, and 100 ng/ml of DNP-HSA for 10 min. The cell morphology of each group was observed under a transmission electron microscope (JEM-1200EX, Japan).

To determine the effects of p38 MAPK on the Nrf2/HO-1 activation, GLA-treated RBL-2H3 cells were pretreated with p38 MAPK inhibitor SB202190 (SB, 20 *µ*M; #8158; Cell Signaling Technology) for 1 h.

To further investigate the relationship between HMGB1 and mast cells, the RBL-2H3 cells were stimulated with 20 ng/ml of rmHMGB1 (Abcam, USA) for 30 min, with or without GLA (5.0 *μ*M). Meanwhile, RBL-2H3 cells were transfected with siRNA (scrambled) (50 nM) or HMGB1 siRNA (50 nM) for 6 h according to the instructions of the transfection reagent Lipofectamine TM2000 (Thermo Fisher Scientific). After siRNA transfection, RBL-2H3 cells were treated with Ag, IgE, or GLA as above described. siRNA was purchased from RIBO Biology (Guangzhou).

### 2.5. MTT

RBL-2H3 or RPMCs cells were seeded in a 96-well plate at 2 × 10^4^ per well and treated with GLA for 24 h. Then, the cells were incubated with 1 mg/mL MTT (Santa Cruz, CA, USA) at 37°C. DMSO (Solarbio, Beijing, China) was added to dissolve toluidine crystals. The absorbance at 570 nm was measured on Spectra MAX PLUS (Molecular Devices).

### 2.6. Determination of Histamine and *β*-Hexosaminidase

RBL-2H3 cells (1 × 10^6^/mL) were sensitized by adding 50 ng/mL anti-DNP IgE. The cells were incubated with or without GLA (0.1 *μ*M, 1.0 *μ*M, and 5.0 *μ*M) for 30 min followed by incubation with 100 ng/mL DNP HSA for 10 min. After centrifugation, the supernatant was taken and histamine content was measured using the radioenzymatic method. The release of *β*-hexosaminidase was detected according to the protocol described previously [12]. Absorbance was measured at 405 nm using a microplate reader (Bio-rad680, USA).

### 2.7. Calcium Intake

RBL-2H3 cells (1 × 10^6^/mL) were incubated with HEPES-Tyrode buffer (except for CaCl_2_ and MgCl_2_) (Sigma, USA) containing 1*μ* Ci^45^Ca^2+^/mL. Then, cells were treated with 50 ng/mL anti-DNP IgE for 6 h. Then, different concentrations of GLA were added for incubation for 30 min at 37°C followed by a challenge with 100 ng/mL DNP-HSA for 10 min. After cell lysis with 10% Triton X-100, radioactivity was determined by a scintillation *β*-counter (Liquid Scintillation Analyzer, Canberra Industries, USA).

### 2.8. ELISA

The levels of TNF-*α*, IL-1*β*, IL-4, IL-8, and IL-13 in the culture medium of RBL-2H3 cells and RPMCs were determined by corresponding ELISA kits (Abcam, USA) according to the manufacturer's instructions.

### 2.9. Western Blot

Nuclear and cytoplasmic proteins were extracted from RBL-2H3 cells. Protein concentration was determined by BCA as previously described [13], and 20 *μ*g of protein was subjected to SDS-PAGE analysis. After 1 h blocking, different primary antibodies (1 : 1000) were added and incubated overnight at 4°C, and different secondary antibodies were incubated for 1 h at 37°C. Antibodies including NF-*κ*Bp65 (#8242), phospho-NF-*κ*Bp65 (#3039), PARP (#9532), I*κ*B*α* (#4814), phospho-I*κ*B*α* (#2859), p38MAPK (#8690), phospho-p38MAPK (#4511), ERK (#4695), phospho-ERK (#4370), JNK (#9258), phospho-JNK (#9255), Syk (#13198), phospho-Syk (#2710), Lyn (#2796), phospho-Lyn (#2731), Gab2 (#3239), phospho-Gab2 (#3881), PLC-*γ*1 (Tyr, #2821), phospho-PLC*γ*1 (#8713), HMGB1 (#3935), TLR4 (#14358), MyD88 (#3699), Nrf2 (#12721), HO-1 (#82206), Keap1 (#8047), *β*-actin (#3700), anti-rabbit IgG(H + L) (#14708), and anti-mouse IgG (H + L) (#14709) were all purchased from Cell Signaling Technology (Beverly, MA, USA). Protein bands were analyzed using an optical density scanner with a Gel Doc XR system (Bio-Rad, USA).

### 2.10. Statistical Analysis

SPSS 23.0 (IBM, Armonk, NY, USA) was used for data analysis. The results were presented as mean ± SEM. Graphs were plotted using Prism 7.0 software (GraphPad, San Diego, CA, USA). One-way analysis of variance followed by Tukey's test for post hoc analysis was used. A *P* value < 0.05 was considered statistically significant.

## 3. Results

### 3.1. GLA Inhibits Ear Swelling in PCA Mice

**Figure 1:**
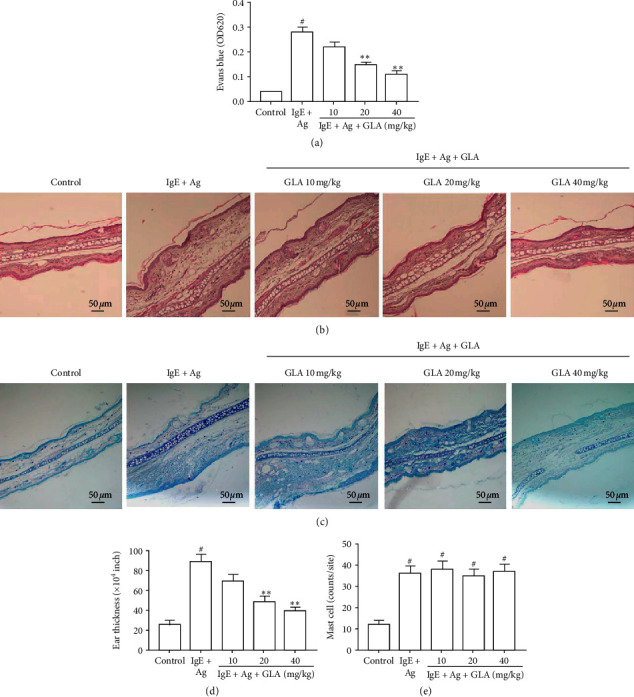
Effect of GLA on anti-DNP-IgE-mediated passive cutaneous anaphylaxis (PCA) mice. (a)The absorbance of dye extravasation on the ears of PCA mice by spectrophotometer. Ear sections were stained with hematoxylin and eosin (b) and toluidine blue (c) (scale bar = 50 *μ*m). (d) Ear thickness comparison. (e) The numbers of dermal mast cells (toluidine blue staining). All data represent the mean ± SEM (*n* = 3). Compared with the control group, ^#^*P* < 0.05. Compared with IgE + Ag group, ^*∗∗*^*P* < 0.01.

In order to evaluate the effect of GLA on acute allergic inflammation *in vivo*, we established a PCA mouse model. Our results showed that GLA inhibited Evans blue extravasation in a dose-dependent manner (Figure 1(a)). The ear thickness increased in the IgE + Ag treatment group, while GLA significantly relieved ear swelling (Figures 1(b) and 1(d)). After staining with toluidine blue, the number of mast cells in the ears of the IgE + Ag group increased significantly more than the control group (Figures 1(c) and 1(e)). However, GLA treatment did not significantly affect the number of mast cells. These results indicate that, during antigen-induced allergy, GLA inhibits mast cell degranulation, thereby reducing the anti-DNP IgE/DNP HSA-mediated PCA response, rather than reducing the number of mast cells.

### 3.2. GLA Reduces Histamine and *β*-Hexosaminidase Release and Prevents Calcium Influx

**Figure 2:**
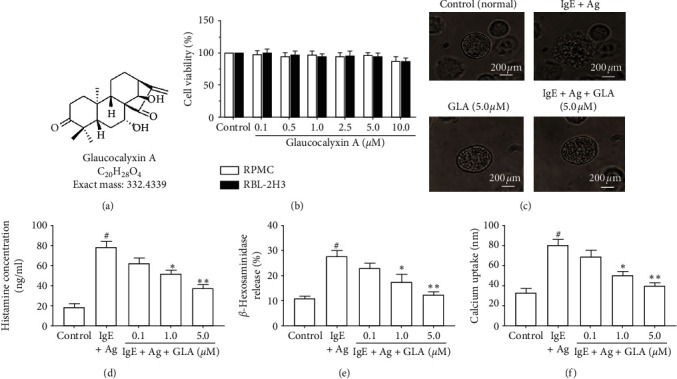
Effect of GLA on cell viability and degranulation in RPMCs and RBL-2H3 cells. (a) Chemical structure of GLA. (b) Measurement of cell viability using MTT assay. RPMCs were divided into control, IgE + Ag (sensitized with 50 ng/mL anti-DNP IgE for 6 h and challenged with 100 ng/ml DNP-HSA) and IgE + Ag + GLA (sensitized with 50 ng/mL anti-DNP IgE for 6 h treated with 5 *μ*g/mL GLA and then challenged with 100 ng/ml DNP-HSA). (c) Morphology changes of degranulation of RPMCs (magnification, ×1,000). (d) Histamine concentration. (e) *β*-Hexosaminidase release. (f) Calcium uptake. All data represent mean ± SEM (*n* = 3). Compared with the control group, ^#^*P* < 0.05. Compared with the IgE + Ag group, ^*∗*^*P* < 0.05 and ^*∗∗*^*P* < 0.01.

The chemical structure of GLA is shown in Figure 2(a). After RPMC and RBL-2H3 cells were treated with different concentrations (0.1–10 *μ*M) of GLA for 24 h, MTT was used to detect cell viability. The results showed that GLA had little effect on the viability of RPMC cells and RBL-2H3 cells (Figure 2(b)). Moreover, Figure 2(c) shows the morphology changes of RPMCs under different GLA treatments. The morphology of normal RPMC was round, with a regular surface and fine particles. After treatment with IgE + Ag, there was degranulation of RPMCs. The cells were swelled and became irregular. However, after GLA treatment, the cell swelling was significantly improved, and the cell size was slightly larger than normal, which significantly inhibited the degranulation of RPMCs. In addition, GLA inhibited the release of histamine (Figure 2(d)) and *β*-hexosaminidase (Figure 2(e)) in RPMC cells (*P* < 0.05). Similarly, intracellular calcium in RPMCs of IgE + Ag group increased rapidly, while GLA significantly inhibited intracellular calcium in a dose-dependent manner (Figure 2(f)). These results indicate that GLA inhibits mast cell degranulation by reducing the release of histamine and *β*-hexosaminidase and preventing calcium influx.

### 3.3. GLA Suppresses Proinflammatory Cytokines and Fc*ε*RI Signaling Pathway

**Figure 3:**
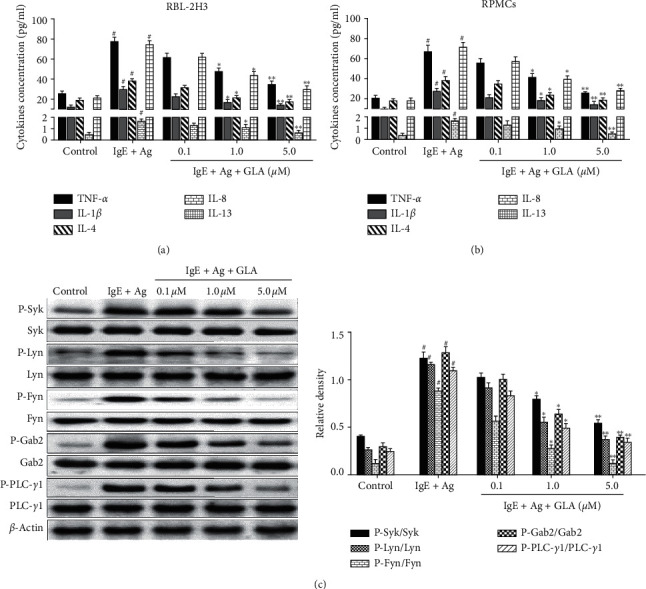
Effect of GLA on the secretion of proinflammatory cytokines and Fc*ε*RI-mediated signaling pathway in RBL-2H3 cells. RPMCs and RBL-2H3 cells were stimulated with anti-DNP IgE (50 ng/ml) for 6 h and challenged with DNP-HSA (100 ng/mL) with or without pretreatment with GLA (0.1, 1.0, and 5.0 *μ*M). Cytokine levels in the supernatants of RPMCs (a) and RBL-2H3 cells (b) were measured by ELISA. (c) The protein levels of Syk, Lyn, Fyn, Gab2, and PLC-*γ*1 and their phosphorylation were determined by Western blot. All data represent the mean ± SEM (*n* = 3). Compared with the control group, ^#^*P* < 0.05. Compared with the IgE + Ag group, ^*∗*^*P* < 0.05 and ^*∗∗*^*P* < 0.01.

It is well known that the activated mast cells release proinflammatory cytokines, leading to allergic inflammation [14]. Therefore, we detected the expression of proinflammatory cytokines in RBL-2H3 cells and RPMC after sensitization by IgE with ELISA. The results showed that compared with the control group, TNF-*α*, IL-1*β*, IL-4, IL-8, and IL-13 in the IgE + Ag group were significantly increased, while GLA significantly inhibited the secretion of cytokines in a dose-dependent manner (Figures 3(a) and 3(b)).

In addition, we analyzed the phosphorylation changes of key proteins in Fc*ε*RI signaling pathway with Western blot. As shown in Figure 3(c), after anti-DNP-IgE/DNP-HSA stimulation, the phosphorylation of Syk, Lyn, Fyn, Gab2, and downstream factor PLC-*γ*1 in RBL-2H3 cells increased significantly, while GLA inhibited protein phosphorylation in a dose-dependent manner (Figure 3(c)). These results suggest that GLA may inhibit mast cell-mediated allergic inflammatory responses through Fc*ε*RI-dependent signaling pathways.

### 3.4. GLA Inhibits Mast Cell Activation through Regulation of p38 MAPK-Stimulated Nrf2/HO-1 Signaling Pathways

**Figure 4:**
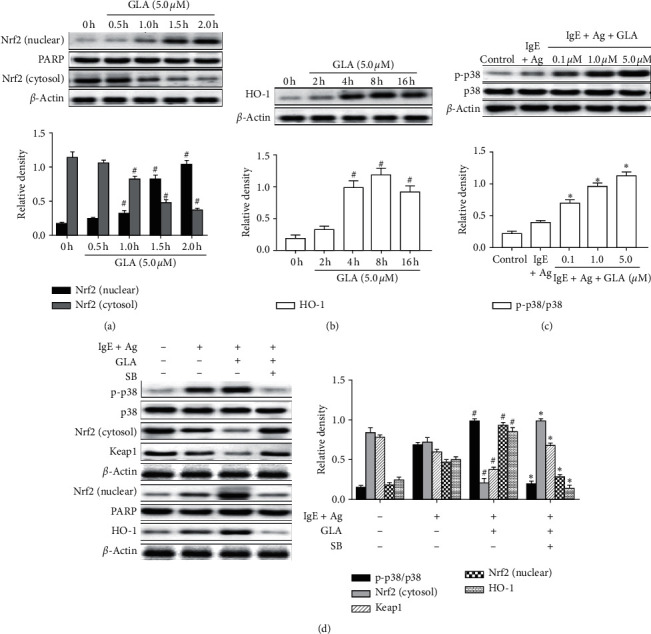
Effect of GLA on the intracellular signal transduction in RBL-2H3 cells. RBL-2H3 cells were stimulated with 50 ng/mL anti-DNP IgE for 6 h and challenged with 100 ng/ml DNP-HSA in the presence or absence of GLA (0.1, 1.0, or 5.0 *μ*M). Protein levels were measured with Western blot. The protein levels of Nrf2 (a) and HO-1 (b). 0 h indicates the stimulation with anti-DNP IgE/DNP-HSA without GLA. (c) The protein levels of p38 MAPK. (d) Protein levels of p38, Nrf2, Keap1, and HO-1. Each data represents the mean ± SEM (*n* = 3). Compared with 0 h, vehicle, or control group, ^#^*P* < 0.05. Compared with IgE + Ag group, ^*∗*^*P* < 0.05.

Studies have shown that mast cell activation could be attenuated via Nrf2/HO-1 signaling pathway [15, 16]. Previous studies have also shown that MAPK is a central pathway involved in Nrf2 activation and translocation [17, 18]. To determine the mechanism underlying the inhibition of GLA on mast cell-mediated allergic inflammation, we tested whether GLA affects the Nrf2/HO-1 signaling pathway. We first checked whether GLA induces Nrf2 upregulation in the RBL-2H3 cell. As shown in Figure 4(a), GLA could increase the nuclear accumulation of Nrf2 under anti-DNP IgE/DNP-HSA stimulation, which was evident at 1 h and peaked at 2 h. In addition, the effect of GLA on HO-1 showed a similar trend to that of Nrf2. However, HO-1 did not increase significantly until 4 h after induction, peaked at 8 h, and lasted until 16 h (Figure 4(b)).

To determine the upstream pathway of Nrf2/HO-1, we further tested protein changes of the p38 MAPK family by Western blot. The results showed that GLA treatment promoted p38 MAPK phosphorylation (Figure 4(c)). We further used a specific chemical inhibitor of p38 MAPK, namely, SB202190 (SB), to assess whether SB pretreatment prevented Nrf2/HO-1 activation. The results showed that Nrf2 nuclear level and Keap1 cytoplasmic level induced by anti-DNP IgE/DNP-HSA stimulation were almost completely blocked by SB, and HO-1 activity was significantly inhibited (Figure 4(d)). These results showed that GLA stimulated p38 MAPK phosphorylation, reversed the degradation of Keap1, and promoted Nrf2 nuclear transfer and subsequent HO-1 activation, all of which supports that GLA induces Nrf2/HO-1 activation via stimulation of p38 MAPK phosphorylation to further inhibit mast cell degranulation.

### 3.5. GLA Downregulates NF-*κ*B Nuclear Translocation by Inhibiting HMGB1/TLR4 Signaling

**Figure 5:**
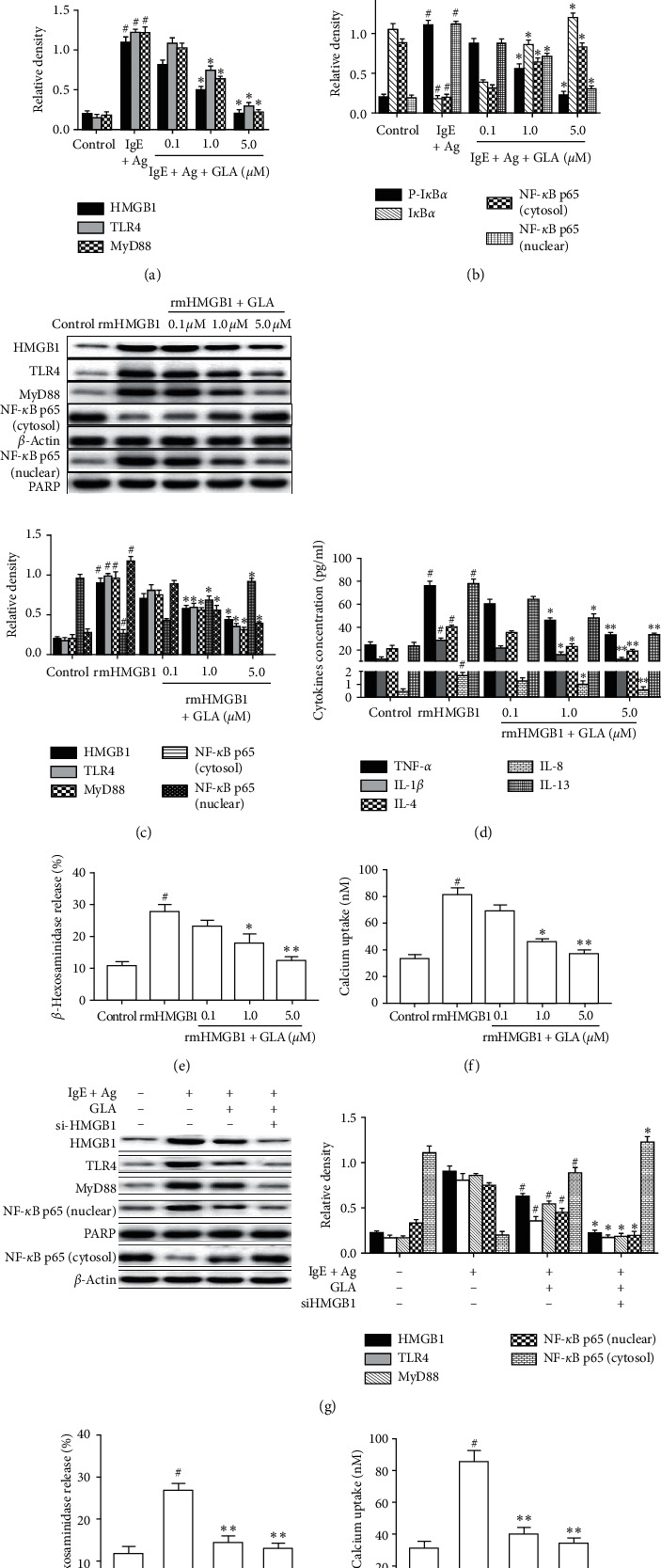
Effect of GLA on HMGB1-mediated signaling pathways in RBL-2H3 cells. Protein levels of HMGB1/TLR4/NF-*κ*B signaling pathway were measure by Western blot. (a) and (b) RBL-2H3 cells were challenged with 100 ng/ml DNP-HSA in the presence or absence of GLA. Protein levels were analyzed. (c) RBL-2H3 cells were stimulated with 20 ng/ml rmHMGB1 with or without GLA (5.0 *μ*M) for 30 min. Protein levels were analyzed. (d) The effect of GLA on proinflammatory cytokines in the rmHMGB1-stimulated RBL-2H3 cells. (e) Release of *β*-hexosaminidase. (f) Calcium uptake was measured by the radioenzymatic method. (g) RBL-2H3 cells were transfected with 50 nM control (siCont) or HMGB1 siRNA (si-HMGB1) for 6 h. After 24 h, they were then stimulated with DNP HSA (100 ng/mL). (h) The release of *β*-hexosaminidase was measured after cell treatment. (i) Calcium uptake was measured after cell treatment. Protein levels were analyzed. Each datum represents the mean ± SEM (*n* = 3). Compared with the IgE + Ag group or rmHMGB1 group, ^#^*P* < 0.05. Compared with IgE + Ag + GLA group, ^*∗*^*P* < 0.05.

Studies have shown that the HMGB1/TLR4 signaling pathway is abnormally activated in mouse models such as asthma and allergic rhinitis [19, 20]. To investigate whether GLA inhibits mast cell activation through the HMGB1/TLR4 signaling pathway, we determined the related protein levels by Western blot. The results showed that HMGB1, TLR4, and MyD88 were significantly increased after activation by anti-DNP IgE/DNP-HSA treatment, and the NF-*κ*B p65 nuclear transfer was enhanced. Interestingly, these changes were all reversed by GLA treatment (Figures 5(a) and 5(b)). HMGB1 can also stimulate mast cell activation [21]. To further investigate the relationship between GLA and HMGB1-induced mast cell activation, we pretreated RBL-2H3 cells with rmHMGB1 and further measured HMGB1, TLR4, and NF-*κ*B p65 by Western blot, as well as detecting calcium influx and related inflammatory factors by ELISA. The results showed that pretreatment of RBL-2H3 cells with rmHMGB1 induced an increase in the activity of HMGB1 and TLR4 and promoted NF-*κ*B p65 transfer into the nucleus after I*κ*B*α* degradation (Figure 5(c)). Moreover, the release of *β*-hexosaminidase and calcium influx was significantly increased, and the release of inflammatory factors (Figure 5(d)), *β*-hexosaminidase (Figure 5(e)), and calcium influx (Figure 5(f)) was significantly increased, indicating that rmHMGB1 could induce mast cell degranulation. After GLA treatment, NF-*κ*B p65 nuclear translocation was significantly inhibited, while NF-*κ*B (cytosol) expression was increased (Figure 5(c)). Additionally, inflammatory factor and *β*-hexosaminidase release were reduced, and calcium influx was inhibited (Figures 5(d)–5(f)).

To further validate the protein level of downstream signaling pathways, we transfected RBL-2H3 cells with HMGB1-specific siRNA. As shown in Figure 5(g), HMGB1-specific siRNA suppressed HMGB1 expression and inhibited their downstream protein TLR4 and MyD88 and the nuclear transportation of NF-*κ*B p65, with a synergistic effect with GLA. Additionally, after si-HMGB1 treatment, *β*-hexosaminidase (Figure 5(h)) and calcium influx (Figure 5(i)) were significantly inhibited.

Taken together, GLA may inhibit mast cell degranulation through the regulation of the HMGB1/TLR4/NF-*κ*B signaling pathway, as well as the nuclear translocation of NF-*κ*Bp65.

## 4. Discussion

Worldwide, the incidence of allergic diseases has increased rapidly [22]. Mast cells play a key role in allergic disease, which could cause anaphylaxis by degranulation [23]. Mast cell degranulation is mediated by IgE/Fc*ε*RI, which leads to the secretion of inflammatory mediators [24]. PCA animal models allow evaluation of mast cell-mediated localized anaphylaxis *in vivo* [12, 23]. In this experiment, we observed that GLA significantly inhibited Evans blue extravasation and ear thickness in PCA mice; however, GLA attenuated the local inflammatory response without affecting mast cell number. The results indicate that GLA has anti-inflammatory effects in the PCA mice.

Fc*ε*RI-mediated mast cell activation involves calcium influx [25]. The release of intracellular calcium leads to the degranulation of mast cells. During degranulation, mast cells secrete a variety of inflammatory mediators, including histamine, *β*-hexosaminidase, cytokines, proteases, chemokines, and growth factors [26]. Histamine is the key mediator of the allergic inflammatory response [27]. In addition, like histamine, *β*-hexosaminidase is also an important biomarker for mast cell degranulation and also triggers an allergic inflammatory response [28]. Herein, we found that cell viability of mast cells was not significantly affected by GLA. Moreover, GLA reduced intracellular calcium levels in a dose-dependent manner and inhibited the release of histamine and *β*-hexosaminidase in mast cells. In addition, proinflammatory cytokines secreted after mast cell activation further promote the progression of the allergic inflammatory response [29, 30]. The key proinflammatory factors include TNF-*α*, IL-4, IL-1*β*, IL-8, and IL-13 [14]. ELISA results showed that GLA inhibited the levels of TNF-*α*, IL-4, IL-1*β*, IL-8, and IL-13 in RBL-2H3 cells and RPMCs induced by anti-DNP IgE in a dose-dependent manner. Therefore, our results indicate that GLA has a strong inhibitory effect on mast cell degranulation but has no significant cytotoxicity on cell viability.

The activation of mast cells depends on the activation of Syk, which is regulated by the interaction of Fc*ε*RI with Lyn and Fyn [31]. Syk plays an important role in regulating the production of inflammatory cytokines in mast cells by stimulating downstream molecules such as Gab2 and PLC-*γ*1 [2]. Activation of these signaling pathways leads to mast cell degranulation and the release of proinflammatory cytokines. Our data found that GLA inhibited antigen-induced phosphorylation of Lyn, Syk, Gab2, and PLC-*γ*1 in a dose-dependent manner.

MAPK plays different roles in different cell types under the same stimulation. It has been reported that induction of phosphorylation of p38 MAPK can mediate the activation of Nrf2/HO-1 [32]. MAPK-mediated Nrf2 activation regulates the expression of HO-1, which has been shown to play an important role in inhibiting excessive production of proline [33]. Phosphorylation of p38 triggers Keap1 degradation and promotes Nrf2 translocation to the nucleus, which then binds to HO-1 promoter, and leads to HO-1 overexpression [15]. Our results showed that pretreatment of RBL-2H3 cells with GLA significantly increased p38 MAPK phosphorylation. Interestingly, the Nrf2/HO-1 signal pathway activated by anti-DNP IgE/DNP-HSA stimulation was reduced by SB, the specific inhibitor of p38MAPK. SB could reduce Keap1 expression and Nrf2 nuclear expression. These results indicate GLA induces Nrf2/HO-1 overexpression by activating p38 MAPK phosphorylation, which then further reduces the allergic inflammatory responses.

HMGB1 is a proinflammatory cytokine, which mediates immune and inflammatory responses through NF-*κ*B signaling pathways [20]. TLR4 activates NF-*κ*B through MyD88 and forms a complex with its nuclear factor inhibitory protein I*κ*Ba into the nucleus to induce immune and inflammatory responses [7]. A recent study has shown that, in animal models of atopic dermatitis, atopic dermatitis-like symptoms were further improved by inhibiting HMGB1 expression in mast cells, preventing NF-*κ*B nuclear translocation, and reducing the release of cytokines such as TNF-*α* and IL-1*β* [21]. Our results showed that treatment of mast cells with anti-DNP IgE/DNP HSA or rmHMGB1 induced mast cell degranulation. GLA treatment resulted in a dose-dependent decrease in HMGB1 and TLR4 expression, reduced the phosphorylated I*κ*B*α* and nuclear NF-*κ*B, and decreased calcium influx and *β*-hexosaminidase release. Finally, treatment of mast cells with siRNA HMGB1 significantly inhibited HMGB1 levels, as well as MyD88 and TLR4. Additionally, siRNA HMGB1 also reduced intracellular calcium levels and inhibited the release of *β*-hexosaminidase. These results confirmed that GLA could inhibit mast cell degranulation by inhibiting the HMGB1/TLR4 signaling pathway and that HMGB1 as a proinflammatory cytokine can induce mast cell activation.

## 5. Conclusions

In summary, GLA inhibits mast cell activation and mediates Nrf2/HO-1 overexpression by activating anti-DNP IgE-induced p38 MAPK phosphorylation. In addition, GLA inhibits HMGB1/TLR4 overexpression and prevents NF-*κ*Bp65 nuclear transfer, thereby reducing allergic inflammation. GLA may be a potential new drug for the treatment of mast cell-mediated allergic diseases.

## Data Availability

The data used to support the findings of this study are available from the corresponding author upon request.
